# Awareness level, knowledge and attitude towards breast cancer among staff and students of Hail University, Saudi Arabia

**DOI:** 10.1371/journal.pone.0282916

**Published:** 2023-03-15

**Authors:** Meshari Almeshari, Yasser Alzamil, Amjad Alyahyawi, Ahmad Abanomy, Omar Althmali, Mamdouh S. Al-Enezi, ShashiKumar C. G., Hamid Osman, Mayeen Uddin Khandaker

**Affiliations:** 1 Department of Diagnostic Radiology, College of Applied Medical Sciences, University of Hail, Hail, Saudi Arabia; 2 Centre for Nuclear and Radiation Physics, Department of Physics, University of Surrey, Guildford, Surrey, United Kingdom; 3 Department of Radiological Sciences, College of Applied Medical Sciences, King Saud University, Riyadh, Saudi Arabia; 4 Department of Physiotherapy, College of Applied Medical Sciences, University of Hail, Hail, Saudi Arabia; 5 Department of Radiological Sciences, College of Applied Medical Sciences, Taif University, Taif, Saudi Arabia; 6 Centre for Applied Physics and Radiation Technologies, School of Engineering and Technology, Sunway University, Bandar Sunway, Kuala Lumpur, Selangor, Malaysia; 7 Department of General Educational Development, Faculty of Science and Information Technology, Daffodil International University, Dhaka, Bangladesh; University of Nigeria Teaching Hospital, NIGERIA

## Abstract

**Introduction:**

Awareness of screening procedures and illness warning signals is critical for expanding and implementing screening programs in society, which would improve the odds of early identification of breast cancer.

**Objectives:**

This study aimed to evaluate the knowledge, awareness, attitudes, and practices related to breast cancer risk factors, signs, symptoms and methods of screening among female faculty and students at Hail University in the Kingdom of Saudi Arabia.

**Methods:**

A cross-sectional study was conducted from January 2021 through February 2021 in the Hail region of Saudi Arabia. A closed-ended questionnaire, which consisted of 37 questions, was distributed online (using a Google Forms link) in both English and Arabic languages. Data was collected from 425 female subjects who participated in the study.

**Results:**

The study showed an overall knowledge level of 46.36% regarding breast cancer. Participants had average knowledge about risk factors, signs, and symptoms, whereas their awareness and practice of breast self-examination and screening methods were weak.

**Conclusion:**

The current study concluded that public awareness of breast cancer remains relatively low, and Saudi Arabia still needs several public awareness initiatives using mass media, such as television, the Internet, and radio, as well as social media. Special awareness programs should also be held in places where a large number of women can easily be reached, such as colleges, universities, and hospitals.

## 1. Introduction

Breast cancer is the most common type of cancer and the second highest cause of mortality in women [1]. The foremost common sort of breast cancer is ductal carcinoma (85–90% of all cases), which starts within the lining of the milk ducts. The other sort of breast cancer is lobular carcinoma (8% of all cases), which starts within the lobules of the breast [1]. Globally, among all types of cancer, breast cancer accounts for 11.7% of all cancer incidents with approximately 2.3 million new cases in women in the year 2020 [2]. Breast cancer incidence is alarmingly high in the Middle East, which could be linked to delayed diagnosis allowing the disease to progress [3].

Historically, the risk of breast cancer among the female population in Saudi Arabia was presumed to be insignificant; nonetheless, updated statistics contradicted that presumption indicating that breast cancer incidences among the Saudi female population is as significantly high as global statistics [4]. Moreover, most cases of breast cancer in Saudi Arabia are diagnosed at a late stage, and premenopausal women below the age of 45 are more likely to be affected than those in Western countries [1].

Gender, age, family history, racial factors, radiation exposure, breast changes, early menarche, late menopause, prolonged null parity, overweight, diet, alcohol consumption, smoking, excessive estrogenic exposure, oral contraceptive use, stress, and anxiety account for only 25 to 40 percent of breast cancer cases [5]. The symptoms of early breast cancer vary from one person to another; those symptoms include pain and swelling of the breast, redness of the skin of the breast or nipple, nipple discharge, nipple erosion and painless lumps [6].

Awareness of screening procedures and illness warning signals is critical to advancing and implementing screening programs in society, which will improve the odds of early detection of breast cancer and thus improve quality of life [7]. It is believed that the late diagnosis of breast cancer is due to low awareness in society and the difficulty of accessing appropriate healthcare facilities [8].

The early detection of breast cancer can be accomplished by establishing proactive screenings for women at risk, encouraging yearly mammograms and raising awareness [9]. Women in Saudi Arabia are being educated about breast cancer through a national awareness campaign [10]. Saudi Arabian Ministry of Health recommends that all women aged 40–50 undergo mammographic screening once a year, and once every two years for those above 50 years. A woman with a positive family history of breast cancer should start mammography screening 10 years before her family member was diagnosed with the disease [10].

When dealing with such a serious disease, it is essential to make sure the public is aware of its aggressiveness and the importance of early detection through known signs, as being proactive leads to increased chances of remission. They should also be aware of the various treatment options to combat breast cancer. Even though there is a guideline from the Ministry of Health, there is a dearth of literature on attitudes, knowledge levels and awareness regarding breast cancer in Saudi Arabia. Thus, the purpose of the present study was to evaluate the knowledge, awareness, attitudes, and practices related to breast cancer risk factors, signs and symptoms and methods of screening among female faculty and students at Hail University in the Kingdom of Saudi Arabia.

## 2. Methods

A total of 425 female students and faculty of 18–50 years of age were recruited from the Hail region for this cross-sectional study that was conducted from January 2021 to February 2021. Furthermore, an informed consent was obtained from each participant before being interviewed through the use of an anonymous questionnaire to maintain the privacy of participants. The questionnaire was distributed among participants using a Google forms link in both Arabic and English format after obtaining the approval of the ethical committee of scientific research at Hail University. Also, all participants in this study were informed of their right to refuse to be part of the study at any time.

The survey had 37 questions divided into seven sections: socio-demographic, sources of breast cancer information, knowledge of breast cancer risk factors, awareness and practice of breast self-examination (BSE), knowledge of signs and symptoms, knowledge of breast cancer screening methods, and perceptions about breast cancer treatment. To validate the questionnaire, a pilot study involving randomly selected 15 participants from the Hail region was carried out. As a result, the questionnaire did not need to be modified. Moreover, all pilot study participants were excluded from the study subjects.

In the questionnaire, each correct response was awarded a score of one, whereas an incorrect answer or ‘don’t know’ response was given a zero. A total score for each participant was calculated by summing the number of correct answers. In addition, the level of knowledge was calculated by summing the scores of all knowledge questions. Out of a maximum score of 100, the results were divided into three main categories, Weak (25–49%), Average (50–74%) and Good (75% and above).

Signs and symptoms were estimated as percentages using SPSS version 28 in addition to socio-demographic data, sources of information, awareness and practices, and understanding of risk factors. The association between these characteristics and total knowledge regarding breast cancer was determined using Chi-square analysis. Ethical approval (No. of research H-2021-223 dated 6/12/2021) was granted by the Research Ethics Committee (REC) at the University of Hail, Hail, Kingdom of Saudi Arabia.

## 3. Results

The study involved a total of 425 individuals. Table 1 shows socio-demographic characteristics such as age, marital status, education level, job, and family history of breast cancer. We discovered that 11.1% of the individuals had a history of breast cancer in their families.

**Table 1:** Socio-demographic characteristics of the respondents.

Characteristic	N = 425	%
Age		
Less than 25	286	67.3
25–39	90	21.2
40 and above	49	11.5
Marital status		
Single	286	67.3
Married	90	21.2
Divorced	49	11.5
Education level		
Diploma	127	29.9
Bachelor	232	54.6
Master	15	3.5
Doctorate	51	12.0
Job		
Student	310	72.9
Administrative	47	11.1
Academic	68	16.0
Family History of BC		
Yes	47	11.1
No	378	88.9
No. of pregnant (married only)		
0	197	46.4
1–3	69	16.2
4 and more	100	23.5
First pregnant		
Less than 30	101	23.8
30–40	16	3.8
40 or more	1	0.2
NA	307	72.2

The most common source of information reported by all students was the awareness campaigns (54.1%), followed by media such as television and the Internet (38.6%). Most of the participants were students who had learned about breast cancer through the Ministry of Health’s university-based breast cancer education programme (Table 2).

**Table 2:** Respondents’ source information about breast cancer.

Source information about BC	Yes	%	No	%
University Education	134	31.5	291	68.5
Tv	164	38.6	261	61.4
Family and friends	71	16.7	354	83.3
Medical Journals	83	19.5	342	80.5
Internet	164	38.6	261	61.4
Awareness campaign	230	54.1	195	45.9
Total average	141	33.16	284	66.84

In the current study, we observed poor overall knowledge about breast cancer in 46.22% of the participants. Participants had average knowledge about risk factors as well as signs and symptoms (57.62% and 52%, respectively), whereas awareness and practice of BSE and screening methods were weak (36.37% and 38.9%, respectively) (Table 3).

**Table 3:** Respondents’ overall knowledge towards breast cancer.

Overall knowledge	N	%	X^2^	p-value
Awareness and practice of BSE	189	36.37	5.198	0.023
Knowledge about risk factors	245	57.62	9.941	0.002
Knowledge level about signs and symptoms	229	52.0	2.562	0.109
Knowledge about Screening Methods for breast cancer	165	38.9	21.235	<0.001
Overall knowledge	207	46.22	9.734	0.033

Weak (score 49–25%), Average (score 50–74%), Good (score ≤75%).

Most of the study participants (92.2%) did not know how to perform a BSE, and 56.7% were aware of the importance of BSE for the early detection of breast cancer. Many of the participants understood that obesity, lack of exercise, smoking and X-rays increase the risk of breast cancer. They also had average knowledge of the signs and symptoms of breast cancer; 79.8%, 63.5% and 53.4% of the participants, respectively, knew that changes in breast and nipple shape, size and colour, lumps in the breast and discharge from the nipple are among the signs and symptoms of breast cancer. Also, 96.9% of the participants knew that early detection of breast cancer leads to improve treatment outcomes (Table 4).

**Table 4:** Respondents’ knowledge on awareness and practice of BSE, risk factors, signs and symptoms and screening methods in breast cancer.

Knowledge of awareness and practice of BSE	Yes	%	No	%
Doing BSE monthly	119	28.0	306	72.0
Never performed BSE	240	56.5	185	43.5
BSE helps early detection of BC	241	56.7	184	43.3
Don’t know how to perform	**392**	**92.2**	**33**	**7.8**
No need to perform BSE	85	20	340	80
Discomfort	90	21.2	335	78.8
fear	156	36.7	269	63.3
**Knowledge about risk factors**				
Refrain from breast feeding	286	67.3	139	32.7
Hormonal contraceptive use	305	71.8	120	28.2
Smoking	**345**	**81.2**	**80**	**18.8**
Obesity and lack of exercise	321	75.5	104	24.5
Late menopause	249	58.6	176	41.4
Early menarche	80	18.8	345	81.2
Family history	287	67.5	138	32.5
Aging	238	56	187	44
X-ray exposure	318	74.8	107	25.2
Increase maternal age at first pregnancy (30 and above)	155	36.5	270	63.5
infertility	110	25.9	315	74.1
**Knowledge level about signs and symptoms**				
Change in shape, size and color of breasts and nipples	**339**	**79.8**	**86**	**20.2**
Lump in the breast/armpit	270	63.5	155	36.5
Nipple discharge	227	53.4	198	46.6
Irritation or dimpling of breast skin	166	39.1	259	60.9
Pain in breast/nipple	210	49.0	215	50.6
Pain in armpit	156	36.7	269	63.3
Prominent veins on the surface of the breast	178	41.9	247	58.1
**Knowledge about Screening Methods for breast cancer**				
Have you ever had a mammogram	46	10.8	379	89.2
Doing mammogram yearly	39	9.2	386	90.8
Early detection of BC helps in the treatment of obtaining better results	**412**	**96.9**	**13**	**3.1**

All participants responded to the perception of breast cancer treatment neutrally; 43.3% of the participants agreed with the statement that women could enjoy a good quality of life after treatment of breast cancer. There were neutral responses to the questions regarding the treatment process, the effectiveness of treatment for the young population, social stigma associated with treatment and loss of self-confidence post treatment (59.5%, 37.4%, 33.9% and 42.36%, respectively) (Table 5).

**Table 5:** Respondents’ perception towards breast cancer treatment.

Perception towards BC treatment	Strong Agree N (%)	Agree N (%)	Neutral N (%)	Disagree N (%)	Strongly disagree N (%)
After treatment from BC, a woman can enjoy a good quality of life	**184(43.3)**	140(32.9)	95(22.4)	5(1.2)	1(0.2)
The treatment is a long and painful process	64(15.1)	80(18.8)	**249(58.6)**	30(7.1)	2(0.5)
Treatment for BC is more helpful for young people	39(9.2)	82(19.3)	**253(59.5)**	34(8)	17(4)
Treatment of BC is embarrassing for the woman	30(7.1)	74(17.4)	**159(37.4)**	104(24.5)	58(13.6)
Treatment of BC results in loss of confidence	22(5.2)	65(15.3)	**144(33.9)**	118(27.8)	76(17.9)
Total average	68(15.92)	88 (20.72)	**180 (42.36)**	58 (13.72)	31 (7.24)

## 4. Discussion

This study was conducted to assess the awareness, knowledge, attitudes and perceptions of female students and staff at a university in the Hail region in Saudi Arabia. The results of this study indicate that educational campaigns were the main source of information about breast cancer (54.1%) compared to 43% from a study conducted at Najran University [11]. This was mainly due to their participation in these campaigns, which are organized annually to improve the public’s awareness of breast cancer through lectures, conferences, videos and other source materials. In addition, these students and faculty members frequently referred to medical journals (19.5%), the internet (38.6%) and media sources such as television (38.6%) to learn more about breast cancer.

When the level of knowledge was tested, the results showed a lack of information about the basic nature of the disease. Most of the participants did not know that breast cancer is essentially an abnormal growth; the majority did not know that breast cancer is a multifactorial disease and that early menarche is the least recognized risk factor, whereas smoking, obesity and physical inactivity are the most recognized risk factors, which is consistent with the studies conducted in Jeddah and Riyadh, respectively [12, 13]. More than half of the participants in the study knew that family history is associated with the risk of developing breast cancer. However, only 11.1% had information about their family’s medical history.

In a study conducted in Riyadh [13], women had perceived a higher risk of contracting breast cancer compared to their peers. This suggests that the level of concern about breast cancer occurrence is different even between neighbouring regions in Saudi Arabia. Studies conducted in several other countries documented that more than 30% of women wait for three months or longer before presenting to a health clinic with breast cancer symptoms [14, 15]. In this study, 96.9% of the participants knew that early detection of breast cancer leads to improve treatment outcomes.

The overall awareness and practice of BSE in this study was 36.37%. However, the decision to practice BSE is complex and influenced by several factors, such as age, occupation, knowledge about breast cancer and awareness of BSE. In the present study, 92.2% of the participants did not know how to perform a BSE despite knowing that it helps in the early detection of breast cancer. These results are consistent with previous studies that investigated breast cancer awareness, knowledge and BSE practices among women and college students in Saudi Arabia [12, 16, 17]. Reasons for not performing BSE included lack of time, lack of trust in their ability to do the method correctly, fear of finding a lump, and humiliation associated with breast manipulation. Lack of education and awareness were among the most prominent reasons given by women in this survey for not doing BSE. Women were also unaware that a mammogram would be performed and reported being fearful of the procedure despite 79.8% knowing that changes in breast shape, size and colour are signs and symptoms of breast cancer.

Participants’ overall perception regarding breast cancer treatment was 46.36%. Approximately, 58.6% of women had little knowledge of breast cancer treatment and felt that it is a lengthy and painful procedure; however, 43.3% of participants understood that after treatment, women could enjoy a good quality of life. The BSE proves to be a simple and inexpensive method that plays an important role in the early detection of breast cancer. Failure to perform BSE is associated with delayed diagnosis and subsequently poor long-term survival rates.

Finally, the survey was done through an online Questionnaire in lieu of personal interviews, which was the safest method to protect participants and researchers from spreading coronavirus. However, the online questionnaire had a few disadvantages that could not be avoided including the biased responses. In addition, the calculation of an accurate response rate was found to be difficult because it was not possible to track the number of subjects who received the questionnaire link and who responded accordingly.

## 5. Conclusion

The present study concluded that public awareness of breast cancer remains relatively low, and Saudi Arabia still needs several public awareness initiatives using mass media, such as television, the Internet, and radio, as well as social media. Special awareness programs should also be held in places where a large number of women can easily be reached, such as colleges, universities, and hospitals. Women should be encouraged to talk about self-examination techniques with their friends at home in order to create awareness about BSE and encourage them to get mammograms at regular intervals. It is expected that this study may help in measuring the awareness and therefore planning an effective solution to enhance the awareness, which may lead to a reduction in mortality rate, improvement in quality of life and survival rate due to early detection of breast cancer.

## Supporting information

S1 File (PDF)
