# Supplementary material for: Awareness level, knowledge and attitude towards breast cancer among staff and students of Hail University, Saudi Arabia
Source: PLoS One. 2023 Mar 15;18(3):e0282916. doi: 10.1371/journal.pone.0282916 (PMC10016680; doi:10.1371/journal.pone.0282916)
Supplement: S1 File — (PDF) [file pone.0282916.s001.pdf]

- **PERSONAL data:**

1 . age group:
- Less than 25 years old
- From 25 to 39 years old
- 40 years and over

2 . Marital status
- Single
- married
- divorced

3. Educational level:
- Diploma
- Bachelor's
- Masters
- Doctorate

4 . Job:
- student
- Administrative
- Academic

5 . Is there anyone in the family who has had breast cancer?
- Yes
- No

- **Data required from married women only**

6 . Pregnancy number:
- nothing
- 1-3
- 4 or more

7 . In your first pregnancy, how old were you?
- Less than 30 years old
- 30-40 years old
- More than 40 years
- NA

- Source of information about breast cancer:

- Awareness campaign
- University education
- Media (TV)
- Family and friends
- Medical journals
- Internet

- **A questionnaire to measure the level of breast cancer awareness**

８. Breast cancer affects:
- women only
- men only
- Both sexes

９. The most common types of cancer among women globally and locally:
- breast cancer
- Lung Cancer
- Colon Cancer

１０. Early diagnosis of breast cancer increases the chances of a better outcome (preserving the breast, saving the patient's life)
- Yes
- No

１１. Symptoms that indicate the possibility of cancer:

- Change in the shape, size and color of the breast
- A lump in the armpit area
- Secretions and fluids from the breast (without breastfeeding)
- Itching with crusty sores on the breast
- Breast pain not related to menstruation or breastfeeding
- Armpit pain not related to menstruation
- The appearance of prominent blood vessels on the breast
- *All of the above symptoms

１２. Does advancing age affect the risk of developing breast cancer in general?

- Increases the possibility of injury
- Reduces the possibility of injury

- no effect
- I do not know

１３. Does not having children affect the risk of breast cancer?

- Increases the possibility of injury
- Reduces the possibility of injury
- no effect
- I do not know

14. Among the main reasons for the increased risk of developing breast cancer after menopause in obese women are:

- lack of movement and activity
-* Increased estrogen levels
- High levels of non-oxidants in the blood

１5. Early detection of breast cancer is possible through:

- Breast self examination
- Clinical breast examination
- Mammogram
- Breast biopsy فحص
- Ultrasound examination
- *Everything mentioned

１6. The best time to do a home breast self-exam is:

- Weekly check
- Monthly check up after menstruation
- Monthly check up before menstruation
- Monthly, on the 7-10th day of the menstrual cycle
- Annual periodic check up

１7. Among the most effective and most prominent methods used globally, which help in the rapid recovery of breast cancer:

- *Early detection
- Treatment in advanced stages
- care and hygiene

18. Among the factors that help reduce the incidence of breast cancer:

- Early pregnancy
- Refer to a specialist when discovering any lump in the breast
- Know the signs and symptoms of breast cancer
- Playing sports
- Performing self-examinations of the breast area
- *All of the above

• A questionnaire to determine the level of knowledge of breast cancer treatment:

19. After being treated for breast cancer, a woman can lead a normal life:

- I totally agree
- I agree
- I do not know
- disagree
- I strongly object

20. The stages of breast cancer treatment are long and painful.

- I totally agree
- I agree
- I do not know
- Disagree
- I strongly object

21. Breast cancer treatment is more appropriate and appropriate for young women:

- I totally agree
- I agree
- I do not know
- Disagree
- I strongly object

22. Women who are treated for breast cancer face embarrassment:

- I totally agree
- I agree
- I do not know
- Disagree
- I strongly object

2 3. Breast cancer treatment leads to a loss of self-confidence:

- I totally agree
- I agrees
- I do not know
- Disagree
- I strongly object

2 4. Do you do a monthly self-examination (home)?

- Yes
- No

2 5. Self-examination (home) for early detection of breast cancer:

- Yes
- No

2 6. I have never done a self-examination (at home):

- Yes, I have never done a self-examination
- No, I've already done that

2 7. I believe that one of the reasons for not doing a breast self-exam is because:

- I don't know how
- No need, it's not important
- Uncomfortable
- The fear
- All the above

2 8. Have you ever had a mammogram?

- Yes, I have already had a mammogram
- No, I've never
